# Effect of Combining Early Chemotherapy with Zhipu Liujunzi Decoction under the Concept of Strengthening and Consolidating Body Resistance for Gastric Cancer Patients and Nursing Strategy

**DOI:** 10.1155/2021/2135924

**Published:** 2021-11-30

**Authors:** Min Wang, Shujuan Wang, Qiang Su, Tian Ma

**Affiliations:** ^1^TCM Dispensing Room, Jinan Municipal Hospital of Traditional Chinese Medicine, Jinan 250012, Shandong Province, China; ^2^Department of Rehabilitation Medicine, Second Affiliated Hospital of Bengbu Medical College, Bengbu 233000, Anhui Province, China; ^3^Health Care and Rehabilitation Ward, Jinan Municipal Hospital of Traditional Chinese Medicine, Jinan 250012, Shandong Province, China

## Abstract

**Objective:**

To explore the clinical efficacy of combining early chemotherapy with Zhipu Liujunzi decoction under the concept of strengthening and consolidating body resistance for gastric cancer patients and nursing strategy.

**Methods:**

The clinical data of 100 patients undergoing radical gastrectomy in our hospital from July 2019 to July 2020 were selected for the retrospective analysis, and the patients were divided into the control group and experimental group according to different treatment methods, with 50 cases in each group. Early chemotherapy after surgery was given to patients in the control group, and on the basis of the aforesaid treatment and under the concept of strengthening and consolidating body resistance, patients in the experimental group took Zhipu Liujunzi decoction and received the nursing strategy, so as to compare their effective rate, adverse reaction rate (ARR), immune function indicators, KPS scores, and nursing satisfaction scores.

**Results:**

After treatment, the experimental group obtained significantly higher objective remission rate (ORR) and disease control rate (DCR) (*P* < 0.05), lower carcinoembryonic antigen (CEA) and carbohydrate antigen 19-9 (CA19-9) levels (*P* < 0.001), higher immune parameters levels (*P* < 0.001), higher KPS scores and lower TCM symptom scores (*P* < 0.001), lower PSQI scores, SAS scores, and SDS scores (*P* < 0.001) and higher nursing satisfaction scores (*P* < 0.001), and lower total accidence rate of toxic side effects (*P* < 0.05) than the control group.

**Conclusion:**

Under the concept of strengthening and consolidating body resistance, combining early chemotherapy with Zhipu Liujunzi decoction is a reliable method for improving the immune function and quality of life for gastric cancer patients with higher safety. Such a strategy greatly reduces the tumor marker levels in patients. Further research will be conducive to establishing a better solution for gastric cancer patients.

## 1. Introduction

Gastric cancer, a malignant tumor of the digestive tract that occurs in the epithelium of the human gastric mucosa, is currently one of the malignant tumors with the highest morbidity and mortality worldwide. Relevant survey data showed [1] that compared to 1990, the number of gastric cancer cases increased by about 356,000 and the number of deaths increased by 96,000 in 2018, Southeast Asia has become the main region with an increase in the absolute number of cases and deaths of gastric cancer, and China was the main country responsible for such increase. With higher rates of recurrence and metastasis, the gastric tumor cells cannot be eliminated fundamentally even with surgical treatment, so early chemotherapy becomes the first choice for treating gastric cancer patients [2–4]. However, clinical studies have confirmed that chemotherapy can damage normal cells while eradicating tumor cells, causing severe clinical toxic side reactions in patients, leading to reduced treatment compliance and affecting the therapeutic efficacy [5]. Investigations found that most cancer patients undergoing chemotherapy suffer from the symptoms such as bad mood and poor sleeping quality, while scientific clinical nursing can effectively alleviate such symptoms. In traditional Chinese medicine (TCM) [6, 7], gastric cancer is classified into the categories such as “heart amassment” and “stomach reflux,” which is related to the malfunction of qi and blood caused by eating irregularity, internal injury of seven emotions, and so on. The concept of “strengthening and consolidating body resistance” holds that the onset of tumor is a systemic disease involving the whole body with Yin-Yang imbalance and excess of pathogenic qi caused by the combination of internal and external factors in the human body; thus, TCM treatment emphasizes removing the pathogenic qi and supporting the vital qi at the same time and believes that reestablishing and restoring the zang-fu viscera function and Yin-Yang balance is the root [8]. Zhipu Liujunzi decoction is a TCM decoction based on the concept of strengthening and consolidating body resistance, and with reasonable prescription, it can exert the efficacy such as invigorating the spleen to activate qi and dispelling dampness to dissipate phlegm [9]. The efficacy of this concept in the treatment of sepsis and cervical cancer has been demonstrated, but relevant studies on gastric cancer are currently lacking [10]. Based on this, the clinical efficacy of combining early chemotherapy and Zhipu Liujunzi decoction under the concept of strengthening and consolidating body resistance and nursing strategy was explored in this study, with the results reported as follows.

## 2. Materials and Methods

### 2.1. General Information

The clinical data of 100 patients undergoing radical gastrectomy in our hospital from July 2019 to July 2020 were selected for the retrospective analysis, and the patients were divided into the control group (35∼70 years old) and the experimental group (34∼71 years old) according to the different treatment methods, with 50 cases in each group. The results of comparing the general information of the patients such as their gender, age, and duration of disease were not statistically significant (*P* > 0.05), see Table 1.

### 2.2. Inclusion/Exclusion Criteria

#### 2.2.1. Inclusion Criteria

① The patients were diagnosed with gastric cancer by imaging and pathology and met the indications for radical gastrectomy, and the clinical manifestations included epigastric pain, marasmus, and anorexia
② The patients were at least 18 years old
③ The patients had no previous history of digestive system diseases
④ The patients had normal heart, lung, and kidney function
⑤ The study was approved by the Hospital Ethics Committee, and the patients joined the study voluntarily and signed the informed consent

#### 2.2.2. Exclusion Criteria

① The patients failed to follow the medical advice for standardized chemotherapy or took Zhipu Liujunzi decoction
② The patients had other malignant tumors
③ The patients were allergic to the drug used in the study
④ The patients had uncontrollable active infection, severe electrolyte imbalance, and other diseases
⑤ The patients received other treatment plans for gastric cancer other than the scheme herein

### 2.3. Methods

Early chemotherapy after surgery was given to the patients in the control group by intravenously infusing 130 mg/m^2^ of oxaliplatin (NMPA Approval No. H20093942; manufacturer: Cisen Pharmaceutical Co., Ltd.; specification: 50 mg) once a day and orally taking 1,500 mg of capecitabine (NMPA Approval No. H20133365; manufacturer: Jiangsu Hengrui Medicine Co., Ltd.; specification: 0.5 g *∗* 10 tablets/box) twice a day for consecutive 14 days.

On this basis and under the concept of strengthening and consolidating body resistance, Zhipu Liujunzi decoction was given and nursing strategy was performed to the patients in the experimental group, with the same early chemotherapy as the control group. The recipe of Zhipu Liujunzi decoction was ginseng, solomonseal rhizome, figwort root, *Ganoderma sinense*, glabrous greenbrier rhizome, appendiculate cremastra pseudobulb and bamboo shavings (15 g each), 30 g of Mongolian milkvetch root, 20 g of large-head atractylodes rhizome, 6 g of villous amomum fruit, 6 g of finger citron, 12 g of dried tangerine peel, and 12 g of ginger processed pinellia. All of the herbs were decocted in water for oral dose once a day for consecutive 2 weeks. Patients in both groups were followed up on an outpatient basis for 1 month after treatment.

Nursing strategy: ① the nursing personnel actively communicated with the patients to gain their trust, satisfied their reasonable demands to the greatest extent, and described successful cases for them to build up confidence in treatment and keep a positive attitude; ② the nursing personnel comprehensively analyzed the patients' weight, serum protein content, and other indicators to determine their nutritional status and establish personalized nutritious dietary for them and informed the patients of drinking more water with a daily water intake of not less than 2,000 ml to increase urine output; ③ the nurses repeatedly advised the patients to take a foot bath with warm water, drink warm milk, or listen to soft music before bed to sleep better; ④ nursing measures for complications: during treatment, the common complication in gastric patients was gastrointestinal reaction, usually manifesting as vomiting and nausea, so the patients could orally take drugs such as ondansetron to prevent the adverse reactions in the gastrointestinal tract.

### 2.4. Observation Indicators

Clinical efficacy: by referring to the Response Evaluation Criteria in Solid Tumors (RECIST) [11], it was considered as complete response (CR) if the tumor disappeared for over a month, partial response (PR) if the tumor lesion was shrunk by 50% and more for over a month, stable disease (SD) if the tumor lesion was shrunk by less than 50% for over a month, and progressive disease (PD) if the tumor lesion was increased by 25% and more for over a month. The objective remission rate (ORR) = (CR + PR)/*n* *∗* 100%, and the disease control rate (DCR) = (CR + PR + SD)/*n* *∗* 100%.

5 ml of fasting venous blood was collected from patients in both groups after treatment, and the levels of carcinoembryonic antigen (CEA) and carbohydrate antigen 19-9 (CA19-9) were measured with an electrochemiluminescence instrument and companion test kits (manufactured: Wuhan SPbio Technology Co., Ltd.) and levels of t-cell subsets CD3^+^, CD4^+^, and CD8^+^ were detected with a flow cytometer (manufactured: Beckman Coulter Business (China) Co., Ltd.; model: DxFLEXs).

The international Karnofsky score (KPS) [12] was adopted to evaluate the quality of life after treatment in both groups, and on a scale of 0–100 points, higher scores indicated a higher quality of life in patients. Referring to the evaluation criteria of the efficacy of TCM [13], the clinical symptoms after treatment in both groups were evaluated, which were mainly divided into the assessment of 15 symptoms including abdominal bloating and pain, with a total score of 45 points (3 points for each symptom) and higher scores indicating more severe clinical symptoms.

The sleep quality of patients in the two groups after treatment was evaluated with the Pittsburgh Sleep Quality Index (PSQI) [14], and on a scale of 0–21 points, higher scores indicated worse sleep quality. The patients' mental state was evaluated with Self-Rating Anxiety Scale (SAS) and Self-Rating Depression Scale (SDS) [15], and on a scale of 0–100 points, lower scores indicated more stable emotion.

The patient satisfaction with clinical nursing was evaluated with the Gastric Cancer Patient Satisfaction with Hospitalization Nursing Questionnaire made by our department, and the hundred-mark system was adopted, with scores not less than 60 points indicating satisfied and scores less than 59 points indicating dissatisfied.

Toxic side reactions: the incidence of clinical toxic side effects after treatment in both groups was counted according to the international WHO criteria for the evaluation of toxic side reactions to chemotherapeutic agents [16]. The specific toxic side reactions included neurotoxic effects, myelosuppression, hepatic dysfunction, and gastrointestinal reaction.

### 2.5. Statistical Processing

In this study, the data processing software was SPSS20.0, the picture drawing software for the data was GraphPad Prism 7 (GraphPad Software, San Diego, USA), items included were enumeration data and measurement data, which were expressed by ($\bar{x}\pm s$) and [n(%)] and examined by the t-test and *X*^2^ test, respectively, and differences were considered statistically significant at *P* < 0.05.

## 3. Results

### 3.1. Comparison of Treatment Results

The ORR and DCR of the experimental group were significantly higher than those of the control group (*P* < 0.05), see Table 2.

### 3.2. Comparison of Tumor Marker Levels after Treatment

**Figure 1:**
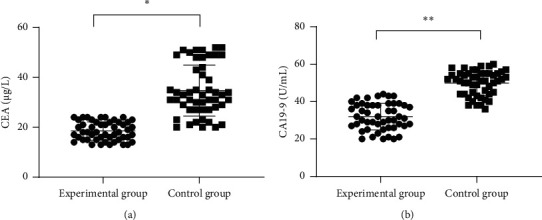
Comparison of tumor marker levels after treatment ($\bar{x}\pm s$). (A) The comparison of CEA levels after treatment in patients between the two groups in *μ*g/L, and the mean CEA levels of the experimental group and the control group were (18.86 ± 2.70) and (34.02 ± 10.01), respectively;  ^*∗*^ indicates that the mean CEA levels after treatment of both groups were significantly different (*t* = 10.340, *P* < 0.001); (B) the comparison of CA19-9 levels after treatment in patients between the two groups in U/mL, and the mean CA19-9 levels of the experimental group and the control group were (32.92 ± 7.30) and (47.22 ± 7.54), respectively; and  ^*∗*^ ^*∗*^ indicates that the mean CA19-9 levels after treatment of both groups were significantly different (*t* = 9.635, *P* < 0.001).

After treatment, the CEA and CA19-9 levels of patients in the experimental group were significantly lower than those in the control group (*P* < 0.001), see Figure 1.

### 3.3. Comparison of Immune Function after Treatment

The immune function of patients was determined by comparing the contents of CD3^+^, CD4^+^, and CD8^+^, and the results showed that the contents of CD3^+^, CD4^+^, and CD8^+^ in patients of the experimental group were significantly higher than those of the control group, which were statistically significant (*P* < 0.05), see Table 3.

### 3.4. Comparison of Clinical Symptoms after Treatment

**Figure 2:**
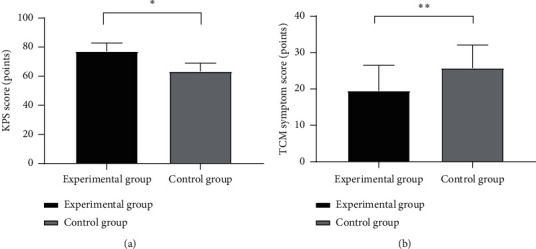
Comparison of clinical symptoms after treatment ($\bar{x}\pm s$). (A) The KPS scores after treatment of the experimental group and the control group, which were (77.13 ± 5.71) and (63.41 ± 5.62), respectively;

After treatment, the experimental group obtained significantly higher KPS scores and lower TCM symptom scores than the control group (*P* < 0.001), see Figure 2.

 ^*∗*^ indicates that the KPS scores after treatment of both groups were significantly different (t = 12.109, *P* < 0.001);

(B) the TCM symptom scores after treatment of the experimental group and the control group, which were (19.53 ± 6.94) and (25.74 ± 6.35), respectively; and  ^*∗*^ ^*∗*^ indicates that the TCM symptom scores after treatment of both groups were significantly different (t = 4.668, *P* < 0.001).

### 3.5. Comparison of Sleep Quality Scores and Metal State Scores after Treatment between the Two Groups

Compared with the control group after treatment, the patients in the experimental group had significantly lower PSQI scores, SAS scores, and SDS scores (*P* < 0.001) and significantly higher nursing satisfaction scores (*P* < 0.001), see Table 4.

### 3.6. Comparison of Incidence Rates of Toxic Side Effects between the Two Groups

The total incidence rate of toxic side effects of the experimental group was significantly lower than that of the control group (*P* < 0.05), see Table 5.

## 4. Discussion

Gastric cancer is a common malignant tumor with the highest prevalence and mortality rate among malignant tumors of the digestive tract, and more and more young people suffer from the disease due to the change of lifestyle and dietary structure. With increasing research [17, 18], people have gradually recognized the pathogenesis of gastric cancer, which is associated with multiple factors. Studies have found that tumor cells are prone to spread and metastasize due to severely impaired cellular and humoral immunity in gastric cancer patients [19]. Currently, surgery is the first choice for the treatment of gastric cancer, but chemotherapy is also necessary because surgery alone cannot completely eradicate tumor cells. However, the lack of targeting of chemotherapy drugs will adversely affect normal cells in the body while killing tumor cells, and more adverse reactions that seriously affect the quality of life may occur in patients after receiving chemotherapy, so an efficient and safe treatment modality is urgently needed [20]. “Strengthening and consolidating body resistance” is a TCM treatment concept, that is, regulating and enhancing the patient's blood and qi level with Chinese herb to elevate immunity and inhibit the growth of tumor cells, thus achieving the goal of treating diseases [21]. Based on this concept, the combination of early chemotherapy and Zhipu Liujunzi decoction was given to the patients in the study, and the results showed that the clinical effect of the experimental group was obviously better than that of the control group, which was due to the fact that herbs such as glabrous greenbrier rhizome and appendiculate cremastra pseudobulb had the effect of detoxification and dissipation blood stasis as well as softening and resolving hard mass, with the efficacy of inhibiting the growth of tumor cells and promoting the tumor cells apoptosis that had been proved in treating the cervical cancer [22].

It has been documented that integrating TCM and Western medicine works well in regulating the body immunity function of patients and is able to kill tumors with the internal and external treatment [23]. The study results showed that various immune parameters of patients in the experimental group were significantly better than those in the control group after treatment, indicating that combining early chemotherapy with Zhipu Liujunzi decoction could improve the immune function and clinical symptoms in gastric cancer patients actively. Investigations and studies found that [24] current chemotherapy treatment only aims to achieve the purpose of tumor lesion shrinkage or disappearance, while the improvement of the patients' body and mental status is often ignored, which leads to a serious reduction in the patients' quality of life, and to a certain extent limits the application range and efficacy of Western medical treatment, resulting in a shorter survival. Therefore, nursing intervention should be intensified during the chemotherapy of gastric cancer patients to improve their mental status and sleep quality, thereby increasing treatment adherence and promoting recovery. In terms of the quality of life improvement, this study found that the combined treatment was better at enhancing the immune function and clinical efficacy than the early chemotherapy alone; thus, the KPS scores of patients in the experimental group were remarkably higher than those in the control group after treatment, indicating the combined treatment could further improve the quality of life in gastric cancer patients. With the nursing strategy, the patients' clinical condition was comprehensively analyzed to establish a feasible nursing plan and guide the nursing practice. The improvement of quality of life was the key for ameliorating the condition of cancer patients [25], so on the basis of early chemotherapy, Zhipu Liujunzi decoction and nursing strategy were adopted under the concept of strengthening and consolidating body resistance, so as to improve various clinical symptoms and then promote the quality of life. However, there were also some shortcomings in this study, for example, failure to do precise dialectical research on patients before the study due to the complex Chinese herbal medicinal ingredients and lack of research on long-term efficacy on gastric cancer patients. Therefore, the physical condition of patients should be accurately and comprehensively evaluated before medication, a targeted treatment scheme should be made by fully considering the condition and treatment will of the patients, and the follow-up time should be prolonged so that more accurate study results can be obtained.

The study results show that combining TCM and chemotherapy can provide a new direction for the treatment of gastric cancer and is of great significance for improving the prognosis of disease. However, deeper studies and exploration are required for further clinical development in the future.

## Figures and Tables

**Table 1:** Comparison and statistics of general information ($\bar{x}\pm s$).

Group		Experimental group	Control group	X^2^/*t*	*P*
Gender (male/female)		23/27	26/24	0.36	0.55
Age (years)		55.32 ± 6.80	55.77 ± 6.39	0.34	0.73
Height (cm)		165.32 ± 9.42	165.64 ± 9.76	0.17	0.87
Weight (kg)		70.83 ± 5.90	70.38 ± 5.64	0.39	0.70
Tumor staging					
Ib		15	17	0.18	0.67
II		26	23	0.36	0.55
III		9	10	0.65	0.42
Pathological type					
Adenocarcinoma		26	28		
Squamous cell carcinoma		24	22		
Histologic grade				0.66	0.42
Poor differentiation		27	31		
Moderate differentiation		23	19		
Albumin (g/L)		30.24 ± 3.17	30.35 ± 3.23	0.172	0.864
Duration of disease (month)		3.68 ± 1.69	3.72 ± 1.73	0.12	0.91
Smoking history (years)		4.31 ± 1.33	4.27 ± 1.38	0.15	0.88
Drinking history (years)		10.96 ± 1.38	10.52 ± 1.22	1.69	0.09
Hypertension (n)		12	10	0.23	0.63
Diabetes (n)		8	7	0.08	0.78
Hyperlipidemia (n)		4	6	0.44	0.51
Educational level	Primary school and below (n)	7	8	0.08	0.78
	Junior high school (n)	15	12	0.46	0.50
	Senior high school (n)	28	30	0.16	0.69
Registration	Rural (n)	33	30	0.39	0.53
	Urban (n)	17	20		
Annual household income	Less than 50,000 yuan	2	4	0.71	0.40
	50,000∼100,000 yuan	26	23	0.36	0.55
	More than 100,000 yuan	22	23	0.04	0.84

**Table 2:** Comparison of treatment results between the two groups [n(%)].

Group	*n*	CR	PR	SD	PD	ORR (%)	DCR (%)
Experimental group	50	17 (34.00)	19 (38.00)	12 (24.00)	4 (8.00)	72.00 (36/50)	92.00 (46/50)
Control group	50	11 (22.00)	15 (30.00)	12 (24.00)	12 (24.00)	52.00 (26/50)	76.00(38/50)
*X* ^2^						4.006	4.762
*P*						<0.05	<0.05

**Table 3:** Comparison of immune function between the two groups (*x* ± *s*, *μ*l^−1^).

Group	CD3^+^	CD4^+^	CD8^+^
Experimental group	843.59 ± 105.77	490.26 ± 87.73	475.00 ± 88.38
Control group	720.26 ± 94.65	332.10 ± 71.19	348.48 ± 74.18
*t*	6.14	9.90	7.75
*P*	<0.001	<0.001	<0.001

**Table 4:** Comparison of sleep quality scores, mental state scores, and nursing satisfaction scores after treatment between the two groups ($\bar{x}\pm s$, scores).

Group	*n*	PSQI	SAS	SDS	Nursing satisfaction scores
Experimental	50	4.27 ± 1.36	27.64 ± 4.27	30.62 ± 5.72	78.24 ± 4.26
Control	50	7.83 ± 1.45	32.16 ± 4.33	36.38 ± 4.86	63.21 ± 4.75
*t*		12.663	5.256	5.426	16.657
*P*		<0.001	<0.001	<0.001	<0.001

**Table 5:** Comparison of incidence rates of toxic side effects [n(%)].

Group	*n*	Neurotoxic effects	Myelosuppression	Abnormal liver function	Gastrointestinal reaction	Total incidence rate
Experimental group	50	0 (0.00)	1 (2.00)	0 (0.00)	2 (4.00)	6.00% (3/50)
Control group	50	2 (4.00)	2 (4.00)	3 (6.00)	3 (6.00)	20.00% (10/50)
*X* ^2^						4.332
*P*						<0.05

## Data Availability

The datasets used and/or analyzed during the current study are available from the corresponding author on reasonable request.
